# Midlife weight gain is a risk factor for obesity-related cancer

**DOI:** 10.1038/s41416-018-0106-x

**Published:** 2018-06-13

**Authors:** Susan Chadid, Martha R. Singer, Bernard E. Kreger, M. Loring Bradlee, Lynn L. Moore

**Affiliations:** 1 Preventive Medicine and Epidemiology, Department of Medicine, Boston University School of Medicine, Boston, MA 02118 USA; 2 General Internal Medicine, Department of Medicine, Boston University School of Medicine, Boston, MA 02118 USA; 3 Framingham Heart Study, National Heart, Lung, and Blood Institute, Framingham, MA 01702 USA

**Keywords:** Cancer epidemiology, Risk factors, Epidemiology

## Abstract

**Background:**

Overweight and diabetes are known cancer risk factors. This study examines independent and combined effects of weight gain and metabolic dysfunction during middle-adult years on obesity-related cancer risk.

**Methods:**

Subjects (*n* = 3850) aged 45–69 years at exams 3–5 in the Framingham Offspring Study were classified according to current and prior (~14 years earlier) weight status, interim weight change and prevalent metabolic dysfunction. Cancer risk among subjects who were overweight at baseline and remained overweight, as well as those who became overweight during follow-up, was compared with risk among normal-weight individuals.

**Results:**

Gaining ≥0.45 kg (≥1.0 pound)/year (vs. maintaining stable weight) over ~14 years increased cancer risk by 38% (95% confidence interval (CI), 1.09, 1.76); combined with metabolic dysfunction, weight gain increased cancer risk by 77% (95% CI, 1.21, 2.59). Compared with non-overweight adults, men and women who became overweight during midlife had 2.18-fold and 1.60-fold increased cancer risks; those who were overweight from baseline had non-statistically significant 28 and 33% increased cancer risks, respectively, despite having a midlife body mass index that was 3.4 kg/m^2^ higher than those who gained weight later.

**Conclusion:**

Midlife weight gain was a strong cancer risk factor. This excess risk was somewhat stronger among those with concurrent metabolic dysfunction.

## Introduction

A number of studies have identified excess body fat as a modifiable risk factor for certain cancers;^1^ fewer studies have examined the effect of *change* in body weight (loss or gain) on obesity-related cancer risk. Amongst these, the relation between weight gain and the risk of postmenopausal breast cancer has been most commonly investigated.^2-4^ Other analyses from the Health Professional’s Follow-up Study demonstrated that weight loss in men decreased the risk of colorectal, pancreatic and oesophageal cancers.^5^ A 2015 meta-analysis found a positive association between weight gain and colorectal cancer.^6^

In terms of overall obesity-related cancer, data from the Women’s Health Initiative (WHI) showed that weight gain increased the risk of obesity-related cancer (a combination of ten cancers).^7^ Results from the Atherosclerosis Risk in Communities study also found that weight gain starting from early adulthood was associated with an increased risk of total cancer, particularly in women.^8^ A recent publication from the Nurses’ Health Study and the Health Professional Follow-Up Study indicated a linear increase of obesity-related cancer associated with increasing weight gain for women.^9^

Components of the metabolic syndrome, such as elevated triglycerides and hypertension, have also been associated with increased cancer risk.^10,11^ The relation between adult weight gain and increased cancer risk may be attributable to intermediate effects on metabolic dysfunction or more direct pro-inflammatory effects of weight gain.

The primary objective of our analyses was to examine the independent and combined effects of adult weight change and accompanying metabolic dysfunction during the middle-adult years on risk of obesity-related cancer. A secondary aim was to examine whether subjects who were overweight or obese prior to middle age had a higher risk of obesity-related cancer than subjects who became overweight or obese later (during middle-adult years). We explored whether these effects were modified by concurrent metabolic dysfunction.

## Methods

### Study population

The Framingham Offspring Study includes the offspring of subjects participating in the original Framingham Heart Study, as well as their spouses. The Offspring Study began in 1971 with the enrolment of 5135 subjects.^12^ Exams 1 and 2 were 8 years apart, with the remaining exams occurring at roughly 4-year intervals. In these analyses, data through exam 8 in 2005–2008 are included. At each examination visit, the following types of data were collected from each participant: anthropometric measures, urinalysis, blood chemistries, blood pressure, medical history and lifestyle habits. At each visit, subjects were asked to report any diseases or conditions that had occurred since their last visit.

Subjects were included in these analyses if they met the following criteria: (1) had baseline weight and height measures; (2) had follow-up weight measures for at least 10 years for determination of weight change (mean weight change period: 14.3 years), (3) were 45–<70 years of age at the end of the weight change period, (4) had complete data for metabolic variables of interest (i.e. high-density lipoprotein-cholesterol (HDL-C), triglycerides, blood glucose and blood pressure) and (5) had complete data for all confounders included in the final models (age, height, physical activity, cigarettes per day, education and alcohol intake). A total of 3850 individuals were included in these analyses.

### Exposure measurements

**Fig. 1:**
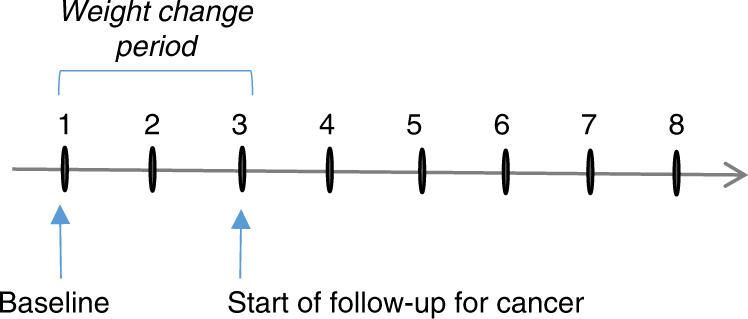
Timeline for data used in the weight change analyses in the Framingham Offspring Study. The interval between exam 1 and 2 was 8 years, while the interval between exams for all subsequent exams was 4 years. For some subjects, baseline was at exam 2 or 3 (if not in age range at exam 1). In this case, the weight change period and cancer follow-up periods were moved forward

Height and weight were measured at each visit using a standard beam balance.^13^ The average of all measures of height prior to age 60 years was used in combination with exam-specific weight measures to calculate exam-specific body mass index (BMI). Using a method previously applied in Framingham, subject-specific slopes for weight change were estimated for each subject by regressing weight on age from baseline to the end of the weight change period.^14^ To be included in the weight change analysis, subjects were required to have a minimum of 10 years of follow-up and three measures of weight. The slopes of weight change were used to classify each subject into one of the following categories: (1) gained ≥0.45 kg/ year (1 pound/year), (2) lost ≥0.45 kg/year or (3) remained weight stable (neither gained nor lost 0.45 kg or more per year). Since subjects were required to have a minimum of 10 years of follow-up, this means that the weight gain group gained at least 4.5 kg during follow-up. The weight stable served as referent group for all analyses. Figure 1 shows the timeline for the measurements described.

For the secondary analyses, subjects were classified according to BMI status at baseline and again at the end of the weight change period (10–20 years after baseline) as shown in Fig. 1. Based on our a priori hypothesis, sensitivity analyses and previous evidence that a BMI between 25 and 30 kg/m^2^ in men and women would reflect different amounts of true body fat and differing levels of metabolic dysfunction, separate cutoff values were used to define overweight in women and men.^15^ Among women, BMI was dichotomised at each time point as <25 vs. ≥25 kg/m^2^. Using BMI status at these two time points, change was classified for women as follows: (a) BMI <25 kg/m^2^ (normal weight) at baseline and normal weight at the end of weight change period, (b) overweight at baseline (BMI ≥25 kg/m^2^) but normal weight by the end of weight change period, (c) normal weight at baseline but overweight by the end of the weight change period and (d) overweight at baseline and remained overweight. Since the rates of obesity-related cancer were the same among subjects in the first two categories above and since very few subjects went from being overweight at baseline to normal weight at follow-up (2% of subjects), these first two groups were combined and used as the referent group for these analyses (called non-overweight middle-aged adults). The other two exposure groups will be identified as those who 'became overweight' (during middle age) and those who were 'consistently overweight' (from younger years into middle age). The same approach was used for men, but with BMI classified as <30 vs. ≥30 kg/m^2^, since this cutoff value for men has been shown to be a more accurate predictor of obesity.^15^

Metabolic dysfunction in these analyses was defined as the presence of two or more of the following risk factors: (a) impaired fasting glucose (IFG) or type 2 diabetes mellitus (T2DM), (b) low HDL-C or elevated triglycerides or taking lipid-lowering medications and (c) high blood pressure (HBP). Prevalent metabolic dysfunction and prevalent IFG or T2DM were cross-classified with weight change to examine potential effect modification by metabolic abnormalities.

In Framingham, IFG and T2DM were diagnosed using the standardised protocols that accounted for uncertain fasting time at exams 1 and 2. T2DM was diagnosed at any exam at which the subject reported using a hypoglycaemia medication (oral or insulin) or if the blood glucose level (fasting or not) was ≥200 mg/dl. Subjects with a glucose level between 126 and 200 mg/dl were diagnosed as having T2DM at that exam if any of the following conditions were met: (a) fasted for 10 h or more, (b) had a history of diabetes or (c) developed definite diabetes at the next exam without gaining weight (7% or more) between exams. At exams 1 and 2, IFG was defined as a glucose level of 126 mg/dl or higher without meeting criteria for T2DM. At all subsequent exams, those who fasted for at least 10 h were considered to have IFG when fasting glucose was 100–125 mg/dl. For some analyses, those with IFG and T2DM were combined to indicate the presence of any glucose dysregulation.

Lipid levels at each exam were derived from blood specimens of subjects fasting at least 12 h using methodology detailed in the previous studies.^16,17^ Plasma HDL-C concentrations were determined through a heparin-manganese chloride precipitation procedure measured using an AA2 Auto Analyzer (Technicon Instruments Corporation, Tarrytown, NY, USA)^18^ described in the Lipid Research Clinics Program.^19^ The plasma HDL-C concentration was determined by subtracting the precipitating portions of LDL and VLDL. Triglycerides were measured through enzymatic methods previously described.^20^ HDL and triglyceride data were used starting at exam 3. A subject was classified as having normal HDL if HDL-C ≥40 mg/dl (men) or ≥50 mg/dl (women) and low HDL if HDL-C <40 mg/dl (men) or <50 mg/dl (women). A subject was classified as having dyslipidaemia if they took lipid-lowering medication, had an HDL-C <40 mg/dl (men), <50 mg/dl (women) or had a triglyceride level ≥150 mg/dl at their baseline exam.^21^

Blood pressure was measured twice with the subject in a seated position.^13^ The mean of two measures for systolic blood pressure (SBP) and diastolic blood pressure (DBP) was used to define HBP status according to the modified JNC-7 criteria as follows: use of medication for hypertension; SBP ≥160 mm Hg or DBP ≥95 mm Hg; or SBP ≥140 mm Hg or DBP ≥90 mm Hg, where SBP was ≥130 mm Hg or DBP was ≥85 mm Hg within the previous two exams.

### Cancer outcomes

Potential cancer cases were first identified through complete medical record review and then subsequently confirmed through pathology, laboratory and/or clinical records.^22^ Cancer diagnosis dates were taken from pathology reports unless diagnosis occurred prior to pathology testing or, in a small number of cases, when pathology results were not available. In the latter case, date of diagnosis was derived from clinical records.^23^ Self-reported cancer diagnoses without pathological or clinical confirmation were not included. Cancer cases were coded using the International Classification of Diseases for Oncology.

Obesity-related cancer outcomes were selected based on previously published studies and included the following: female reproductive (postmenopausal breast, uterine/endometrial and ovarian), colon, rectum, stomach, liver, gallbladder, pancreas, kidney, thyroid, oesophageal adenocarcinoma, leukaemia, non-Hodgkin lymphoma and multiple myeloma.^1,24^ Cases of cancer arising from the uterine cervix were excluded due to its association with human papilloma virus. There were a total of 310 obesity-related cancers included in these analyses.

For colorectal cancer, tumours in the proximal and distal colon were included. Proximal colon cancer included cancer in the caecum, ascending colon, hepatic flexure, transverse colon and splenic flexure. Distal colon cancer included cancer in the descending and sigmoid colon. Appendiceal carcinomas were excluded. Female breast cancer excluded tumours in the skin of the breast.

### Statistical analyses

The rates of obesity-related cancer were calculated for each weight change and weight status change exposure category. Person-years of follow-up time were calculated from the end of the weight change period to the first of the following events: occurrence of an obesity-related cancer, loss to follow-up, date of last exam or date of death. Cancer incidence per 1000 person-years was calculated by dividing the number of obesity-related cancers by the total number of person-years in a given exposure category. Cox proportional hazards were used to estimate hazards ratios for the occurrence of the first obesity-related cancer. The proportional hazards assumptions were tested in all models, and no violations of the assumption were found.

To estimate the independent effect of weight change on obesity-related cancer, all analyses controlled for age (years), sex, cigarettes per day, grams of alcohol per week, average adult height (inches), education (dichotomised as some college or more vs. less) and physical activity. A physical activity index was created by summing self-reported hours of moderate and vigorous activity per day with each type of activity being weighted by the oxygen consumption required for that activity. Factors, such as parity, that were not confounders of the relationship between weight change and cancer risk were excluded from the multivariable models. While time-varying covariates were explored by including changing risk factors such as activity, these risk behaviours were generally stable (thus leading to no changes in the estimated effects). To address concerns about competing risks, we also explored interim development of cardiovascular disease in the models. There was no evidence of bias introduced by competing risks in these analyses.

## Results

Table 1 shows that 145 men and 90 women lost at least one pound per year for at least 10 years; these subjects had a higher mean baseline BMI and were on average slightly older. Those who gained at least a pound per year were slightly younger at baseline. A higher percentage of those who lost weight had T2DM at baseline. The mean weight loss for men in the weight loss category was 0.72 kg (1.6 pounds)/year, while for women it was 0.77 kg (1.7 pounds)/year. Mean weight gain (in the weight gain category) was 0.81 kg (1.8 pounds)/year for men and 0.80 kg (2.0 pounds)/year for women.

**Table 1:** Characteristics according to average yearly weight change

	Men			Women		
	Lost ≥0.45 kg/years (*n* = 145)	Weight stable^a^ (*n* = 1168)	Gained ≥0.45 kg/years (*n* = 530)	Lost ≥0.45 kg/years (*n* = 90)	Weight stable (*n* = 1112)	Gained ≥0.45 kg/years (*n* = 805)
Baseline^b^						
Age (years), mean (s.d.)	41.1 (7.6)	38.5 (8.0)	35.9 (7.6)	42.7 (6.4)	38.3 (7.9)	35.6 (7.1)
BMI (kg/m^2^), mean (s.d.)	29.2 (4.1)	26.7 (3.2)	26.4 (3.8)	30.2 (7.3)	23.6 (4.0)	24.2 (4.5)
Weight, baseline (kg), mean (s.d.)	89.2 (13.3)	81.6 (11.3)	81.1 (12.9)	79.1 (20.5)	61.1 (10.7)	63.5 (11.9)
Start of follow-up (end of weight change period)						
Age (years), mean (s.d.)	54.2 (6.7)	52.6 (6.6)	50.7 (5.9)	55.7 (6.0)	52.5 (6.6)	50.5 (5.6)
BMI (kg/m^2^), mean (s.d.)	26.1 (3.8)	27.0 (3.2)	30.4 (4.5)	26.4 (6.4)	24.3 (3.9)	29.4 (5.6)
Alcohol intake (g/week), mean (s.d.)	19.0 (32.4)	17.9 (22.2)	17.0 (21.8)	7.3 (15.1)	8.5 (12.9)	7.2 (12.6)
Cigarettes per day, mean (s.d.)	9.4 (15.9)	6.3 (12.7)	6.2 (13.2)	8.3 (13.2)	5.4 (10.5)	4.7 (10.5)
Physical activity index, mean (s.d.)	13.3 (8.9)	13.7 (9.8)	12.56 (8.9)	10.1 (7.5)	12.2 (7.4)	11.8 (7.5)
Weight change, kg/year, mean (s.d.)	−0.73 (0.25)	0.06 (0.23)	0.82 (0.37)	−0.78 (0.40)	0.12 (0.22)	0.93 (0.46)
Education (>HS), *n* (column %)	77 (53.1)	745 (63.8)	365 (68.9)	37 (41.1)	623 (56.0)	446 (55.4)
T2DM, *n* (column %)	27 (18.6)	59 (5.1)	32 (6.0)	15 (16.7)	27 (2.4)	30 (3.7)
Dyslipidemia, *n* (column %)	59 (40.7)	597 (51.1)	329 (62.1)	41 (45.6)	387 (34.8)	417 (51.8)
HBP, *n* (column %)	59 (40.7)	467 (40.0)	233 (44.0)	47 (52.2)	307 (27.6)	240 (29.8)

*BMI* body mass index, *HBP* high blood pressure, *HS* high school, *T2DM* type 2 diabetes mellitus.

^a^Weight stable indicates weight loss or gain of <0.45 kg/year.

^b^Baseline is at the beginning of weight change period

After controlling for confounding by age, height, education level, cigarettes/day, alcohol intake, physical activity, BMI at the start of the weight change period and age at the start of the weight change period, men and women who gained at least a pound per year had a higher risk of obesity-related cancer than those who had stable weight (hazard ratio (HR) 1.32; 95% confidence interval (CI), 0.88, 2.00 for men; HR 1.39; 95% CI, 1.03, 1.87 for women). There was no effect of weight loss on cancer risk (Table 2).

**Table 2:** Risk of obesity-related cancers according to category of weight change

Weight change^a^	*N*	PY	Cases	I/1000 py	HR (95% CI)^b^
All subjects					
Weight loss	235	3631	20	5.51	1.05 (0.65, 1.69)
Weight stable	2280	35,901	173	4.82	1.00
Weight gain	1335	19,180	117	6.1	1.38 (1.09, 1.76)
Men					
Weight loss	145	2210	12	5.43	1.17 (0.62, 2.18)
Weight stable	1168	18,274	74	4.05	1.00
Weight gain	530	7568	34	4.49	1.32 (0.88, 2.00)
Women					
Weight loss	90	1421	8	5.63	0.87 (0.41, 1.84)
Weight stable	1112	17,627	99	5.62	1.00
Weight gain	805	11,613	83	7.15	1.39 (1.03, 1.87)

*CI* confidence interval, *I/1000 py* incidence of cancer cases per 1000 person-years, *HR* hazards ratio.

^a^Weight loss = loss of 0.45 kg or more per year, weight gain = gain of 0.45 kg or more per year, weight stable = loss or gain of <0.45 kg/year.

^b^Adjusted for sex (for all subjects model), age, average adult height, education, cigarettes per day, alcohol intake, physical activity, and BMI at baseline

Table 3 explores the independent and combined effects of weight gain and metabolic dysfunction on obesity-related cancers; subjects who lost weight or had stable weight were combined into a single group. Subjects with stable weight or weight loss and no metabolic dysfunction (defined as two or more metabolic abnormalities) served as the referent group for all analyses. Weight gain in combination with metabolic dysfunction led to a 77% increased risk of obesity-related cancer (95% CI, 1.21, 2.59), while weight gain alone led to a 31% increased risk (95% CI, 1.00, 1.71). Those with stable weight (or who lost weight) who also had two or more metabolic problems had a non-statistically significant 21% increased risk of obesity-related cancer.

**Table 3:** Risk of obesity-related cancer according to combined effect of weight change and MetDys

Weight change^a^/MetDys	*N*	PY	Cases	I/1000 py	HR (95% CI)^b^
All subjects					
Weight loss or weight stable/no MetDys	2131	33,908	161	4.75	1.00
Weight gain/no MetDys	1040	15,219	85	5.59	1.31 (1.00, 1.71)
Weight loss or weight stable/MetDys	384	5623	32	5.69	1.21 (0.83, 1.78)
Weight gain/MetDys	295	3962	32	8.08	1.77 (1.21, 2.59)
Men					
Weight loss or weight stable/no MetDys	1047	16,716	66	3.95	1.00
Weight gain/no MetDys	385	5681	22	3.87	1.15 (0.71, 1.87)
Weight loss or weight stable/MetDys	266	3767	20	5.31	1.25 (0.76, 2.08)
Weight gain/MetDys	145	1887	12	6.36	1.85 (1.00, 3.44)
Women					
Weight loss or stability/no MetDys	1084	17,192	95	5.53	1.00
Weight gain/no MetDys	655	9537	63	6.61	1.34 (0.97, 1.85)
Weight loss or stability/MetDys	118	1856	12	6.47	1.09 (0.60, 1.99)
Weight gain/MetDys	150	2075	20	9.64	1.74 (1.07, 2.82)

*CI* confidence interval, *HR* hazards ratio, *I/1000 py* incidence of cancer cases per 1000 person-years, *MetDys* metabolic dysfunction.

^a^Weight loss =  loss of 0.45 kg or more per year, weight gain = gain of 0.45 kg or more per year; weight stable = loss or gain of <0.45 kg/year.

^b^Adjusted for sex (for all subjects model), age, average adult height, education, cigarettes per day, alcohol intake and physical activity

To determine whether T2DM or IFG might be a stronger predictor of cancer risk than metabolic dysfunction as defined by any two or more metabolic abnormalities, we examined the independent and combined effects of T2DM or IFG and weight gain (Supplemental Table 1). Here, in the absence of weight gain, there was no increased risk of obesity-related cancer associated with prevalent IFG or T2DM. The highest cancer risks were found in both men and women with prevalent IFG or T2DM who had gained more than a pound per year during the weight change period (HR, 1.64; 95% CI, 1.17, 2.28 for men and women combined).

Table 4 shows the BMI status change over the weight change exposure period. Men with a BMI of 30 kg/m^2^ or higher were considered overweight, while women with a BMI of 25 kg/m^2^ or higher were considered overweight. In the first BMI status category, men who were 'not overweight' had a mean BMI of 25.6 kg/m^2^ at baseline and a BMI of 26.2 kg/m^2^ at the end of the weight change period, reflecting some weight gain but not enough to change their classification status. Women in the same category had a baseline BMI of 21.5 kg/m^2^ and a subsequent BMI of 22.4 kg/m^2^. Compared with the 'not overweight' group, men who became overweight had a 2.18-fold increased risk of obesity-related cancer; women who became overweight had a 1.60-fold increased cancer risk. In contrast, both men and women who were already overweight at baseline and remained overweight throughout the weight change period (over an average of 14.3 years) had about a 30% increased risk of cancer. It is also evident in this table that, despite having a higher cancer risk, those who became overweight had a lower BMI at the end of the weight change period than those who were previously overweight and remained so throughout the exposure period.

**Table 4:** Risk of obesity-related cancer in men and women according to change in weight status during middle age

Weight status change	*N*	Baseline BMI^a^	Follow-up BMI^a^	HR (95% CI)^b^
Men				
Did not become overweight^c^	1389	25.6	26.2	1.00
Became overweight^d^	206	27.8	32.0	2.18 (1.33, 3.56)
Sustained overweight^e^	248	32.9	34.4	1.28 (0.76, 2.14)
Women				
Did not become overweight^c^	970	21.5	22.4	1.00
Became overweight^d^	468	22.9	27.8	1.60 (1.12, 2.28)
Sustained overweight^e^	569	29.6	32.2	1.33 (0.94, 1.88)

*BMI* body mass index, *CI* confidence interval, *HR* hazards ratio.

^a^Mean BMI at baseline and at the end of weight change period (average follow-up of 14 years).

^b^Adjusted for age, average adult height, education, cigarettes per day, alcohol intake and physical activity.

^c^Women with BMI <25 kg/m^2^ and men with BMI <30 kg/m^2^ at baseline who had no change in BMI category over ~14-year weight change period.

^d^Women with BMI <25 kg/m^2^ at baseline who had BMI ≥25 kg/m^2^ at the end of weight change period; men with BMI <30 kg/m^2^ at baseline who developed BMI ≥30 kg/m^2^ at the end of weight change period.

^e^Women with BMI ≥25 kg/m^2^ and men with BMI ≥30 kg/m^2^ at baseline who remained overweight throughout weight change period

The final table (Table 5), which combines male and female subjects, examines whether metabolic dysfunction modifies the effect of individual changes in BMI status. Once again, it is evident that those who became overweight had higher risks of obesity-related cancer than those who were already overweight at baseline. Subjects with metabolic dysfunction who became overweight had more than twice the risk of obesity-related cancer than those who were not overweight and did not have metabolic dysfunction. Even without metabolic dysfunction, those who became overweight had a 72% increased risk of cancer (95% CI, 1.26, 2.36).

**Table 5:** Risk of obesity-related cancer according to combined category of weight status change and metabolic dysfunction

Weight status change^a^/metabolic dysfunction	*N*	Baseline BMI^a^	Follow-up BMI^a^	PY	Cases	I/1000 py	HR (95% CI)^b^
Not overweight^c^/without MetDys	2063	23.7	24.4	32,269	136	4.21	1.00
Not overweight/with MetDys	296	25.5	26.2	4146	21	5.06	1.24 (0.78, 1.98)
Became overweight^d^/without MetDys	550	24.3	28.8	8003	58	7.25	1.72 (1.26, 2.36)
Became overweight/with MetDys	124	25	30.3	1677	14	8.35	2.05 (1.18, 3.55)
Sustained overweight^e^/without MetDys	558	29.9	32.2	8855	52	5.87	1.21 (0.87, 1.69)
Sustained overweight/with MetDys	259	32.1	34.4	3762	29	7.71	1.58 (1.05, 2.38)

*CI* confidence interval, *HR* hazards ratio, *I/1000 py* incidence of cancer cases per 1000 person-years, *MetDy* metabolic dysfunction.

^a^Mean BMI at baseline and at the end of weight change period (average follow-up of 14 years).

^b^Adjusted for age, sex, height, education, cigarettes per day, alcohol intake and physical activity.

^c^Women with BMI <25 kg/m^2^ and men with BMI <30 kg/m^2^ at baseline who had no change in BMI category over ~14-year weight change period.

^d^Women with BMI <25 kg/m^2^ at baseline who had BMI ≥25 kg/m^2^ at the end of weight change period; men with BMI <30 kg/m^2^ at baseline who developed BMI ≥30 kg/m^2^ at the end of weight change period.

^e^Women with BM ≥25 kg/m^2^ and men with BMI ≥30 kg/m^2^ at baseline who remained overweight throughout the weight change period

## Discussion

In this prospective study of middle-aged men and women who either gained weight (≥0.45 kg/year) or became overweight or obese (based on change in BMI status) over an average of about 14 years had an increased risk of obesity-related cancer compared with those who had stable weight. The adverse effect of weight gain on cancer risk was stronger among those who also had metabolic dysfunction. There does appear to be some degree of effect modification of weight gain on cancer risk by prevalent metabolic dysfunction.

To better understand the effect of weight gain apart from the amount and duration of excess weight, BMI status at the beginning and end of the weight gain period was considered. Men and women whose weight gain was such that they became overweight over more than a decade of follow-up had greater risks of obesity-related cancer than those who were already overweight at baseline and remained that way throughout. Further, those who became overweight over the ensuing decade had an excess cancer risk even in the absence of metabolic dysfunction. The adverse effect of weight gain was not explained by having a higher absolute BMI level at the end of follow-up, since those who became overweight actually had a lower mean BMI both at baseline and at the end of the weight change period than those who were persistently overweight.

The cancer-promoting effects of weight gain, particularly at older ages, may be distinct from those of BMI. Cellular ageing is a stress response that may protect against cancer development earlier in life, but that may promote potentially cancerous hyperplasias in middle-aged and older adults.^25^ This process of cellular ageing is pro-inflammatory and associated with the release of cytokines, chemokines, and growth factors which in combination with weight gain, also a pro-inflammatory state, may induce the multi-step cancer process. It has also been shown in randomised controlled trials that intentional weight loss is associated with reductions in C-reactive protein, tumour necrosis factor-α, and interleukin-6.^26,27^ It is possible that the cumulative impact of various pro-inflammatory factors on carcinogenesis may be more evident during times of weight gain than during times of weight stability, even when the individual is already overweight.

Some studies have examined the relation between weight or BMI change and overall obesity-related cancer risk and our results are consistent with some of these. One such study found that for every 5% increase in weight from age 25 years to middle-adult years (ages 45–64 years), there were small (3–7%) increases in risk of obesity-related cancers.^8^ However, for every an increase in BMI of 5 kg/m^2^, there was an associated 38% increased risk of obesity-related cancer. In the Health Professional’s Follow-up Study, weight gains of 10–14.9 and ≥ 15 kg (vs. <2.5 kg) from age 21 to ages 40–75 years were associated with 16 and 46% higher risks, respectively, of obesity-related cancer (colorectal, renal, pancreatic and oesophageal) in men.^5^ An analysis from the WHI found a statistically significant 7% increased risk of obesity-related cancer over 10 years among overweight subjects.^7^ Most recently, analyses of data from the Nurses’ Health Study reported a linear increase in obesity-related cancer associated with increasing amounts of weight gain.^9^

The most common obesity-related cancer among women in our analysis was postmenopausal breast cancer, a cancer that is the most frequently studied obesity-related cancer in general.^2–4,28–33^ Data from the National Institutes of Health-AARP Diet and Health Study found that weight gain during several different periods of life (i.e. young adult years, later reproductive years, postmenopausal years) was associated with increased risks of postmenopausal breast cancer,^2^ while a cohort of Norwegian women found that weight gain during the premenopausal and perimenopausal periods (but not the postmenopausal years) was associated with increased risks of postmenopausal breast cancer.^34^ These results are consistent with our results among women who were in their late 30s on average at the beginning of the weight change period. Lifetime weight gain among women in the original Framingham cohort was also associated with increased risk of later-onset breast cancer.^35^ Finally, a meta-analysis of weight gain and individual obesity-related cancers found that for every 5 kg increase in weight there was a statistically significant 11% increased risk of postmenopausal breast cancer (among non-users of hormone replacement therapy (HRT)), a 13% increased risk of ovarian cancer and a 39% increased risk of postmenopausal endometrial cancer.^36^ This same study found a statistically significant 6% increased risk of colon cancer in men for every 5 kg gain in weight. Prior studies of weight change and colon cancer risk have shown mixed results, especially in women.^6,37–40^ Finally, another 2015 meta-analysis found an increased risk for endometrial cancer associated with weight gain, regardless of HRT use.^41^

There are a number of important strengths of this study, including the availability of repeated weight measurements rather than self-reported weight, allowing for a more stable and unbiased estimate of weight change. In addition, there was extensive and careful systematic follow-up for the occurrence of cancer among study subjects, minimising the likelihood of both differential and non-differential misclassification of the outcome. The results are also strengthened by the long-term follow-up for cancer occurrence and the detailed measurement of many important potential confounders. A shortcoming in this study is the limited power in some exposure categories, especially when stratifying by metabolic dysfunction. The absence of data on HRT use and oestrogen receptor status for breast cancers among women is another study limitation.

Previous studies have debated the existence of the 'metabolically healthy obese phenotype'.^42–44^ This raises the question of whether chronic inflammation associated with metabolic dysfunction is separate from the pro-inflammatory effects of fat gain and, additionally, whether individual markers of metabolic health (e.g. HDL, triglycerides, blood pressure, glucose) may function differently as modifiers of the effect of weight gain on cancer risk. In these analyses, individual obesity-related cancers varied in frequency and type between men and women. These analyses combined obesity-related cancers into a single outcome, which could mask the independent effects of weight gain and/or metabolic dysfunction on certain cancers. Despite these limitations, this study demonstrates that weight gain during the middle-adult years is an important risk factor for obesity-related cancers, independent of metabolic dysfunction and independent of the level of BMI itself.

## Electronic supplementary material

Supplemental Table
